# Chlorophyll Fluorescence Video Imaging: A Versatile Tool for Identifying Factors Related to Photosynthesis

**DOI:** 10.3389/fpls.2018.00055

**Published:** 2018-01-30

**Authors:** Thilo Rühle, Bennet Reiter, Dario Leister

**Affiliations:** Plant Molecular Biology, Department of Biology, Ludwig Maximilian University of Munich, Munich, Germany

**Keywords:** photosynthesis, Arabidopsis, Chlamydomonas, chloroplast, screening, chlorophyll fluorescence, forward genetic screen, reverse genetic screen

## Abstract

Measurements of chlorophyll fluorescence provide an elegant and non-invasive means of probing the dynamics of photosynthesis. Advances in video imaging of chlorophyll fluorescence have now made it possible to study photosynthesis at all levels from individual cells to entire crop populations. Since the technology delivers quantitative data, is easily scaled up and can be readily combined with other approaches, it has become a powerful phenotyping tool for the identification of factors relevant to photosynthesis. Here, we review genetic chlorophyll fluorescence-based screens of libraries of Arabidopsis and Chlamydomonas mutants, discuss its application to high-throughput phenotyping in quantitative genetics and highlight potential future developments.

## Introduction

Since the discovery of the rapid fluorescence transient associated with the initial exposure of dark-adapted leaves to light (the Kautsky effect) in 1931, chlorophyll (Chl) fluorescence has emerged as an indispensable probe in photosynthesis research. There are several reasons for this remarkable development: (i) measurements of Chl fluorescence dynamics can be carried out on intact plants or algal cell cultures in an essentially non-invasive manner, (ii) multiple quantitative photosynthetic parameters can be extracted in short measuring times, (iii) Chl fluorescence measurements can be easily combined with other analytical tools, (iv) instrumentation capable of automated quantification and analysis of Chl fluorescence is now commercially available to a broad range of plant scientists and the technique is no longer restricted to a small group of experts, (v) technical advances achieved in recent decades now permit investigations from the single-cell level (Oxborough and Baker, 1997; Küpper et al., 2000; Tseng and Chu, 2017) to crop plants in the field (Virlet et al., 2017), and open up numerous applications, such as the use of Chl fluorescence-derived parameters as indicators of abiotic (Baker, 2008; Rungrat et al., 2016) or biotic stress (reviewed in: Chaerle et al., 2009). A particularly important technological breakthrough in this field was the development of video imaging systems (Omasa et al., 1987; Fenton and Crofts, 1990), which not only paved the way for the examination of the spatial heterogeneity within a sample, but also made it possible to assess large numbers of samples (e.g., individual plants or cell colonies) in a single experimental run. Thus, Chl fluorescence video imaging (CFVI) can be regarded as an ideal phenotyping technology for the identification of mutants affected in photosynthesis.

In the following, we will give an overview of CFVI-based screens which have been carried out on plant and green algal mutant libraries in the past, discuss recent progress and consider how the technology may be further developed in the future. Technical and theoretical aspects of Chl fluorescence imaging have been described in detail in Nedbal and Whitmarsh (2004), as well as in Oxborough (2004). The interested reader is also referred to several excellent review articles for introductions to the biophysical basis and biochemical implications of Chl fluorescence-derived photosynthetic parameters (Maxwell and Johnson, 2000; Roháček, 2002; Baker, 2008; Kalaji et al., 2017).

**FIGURE 1:**
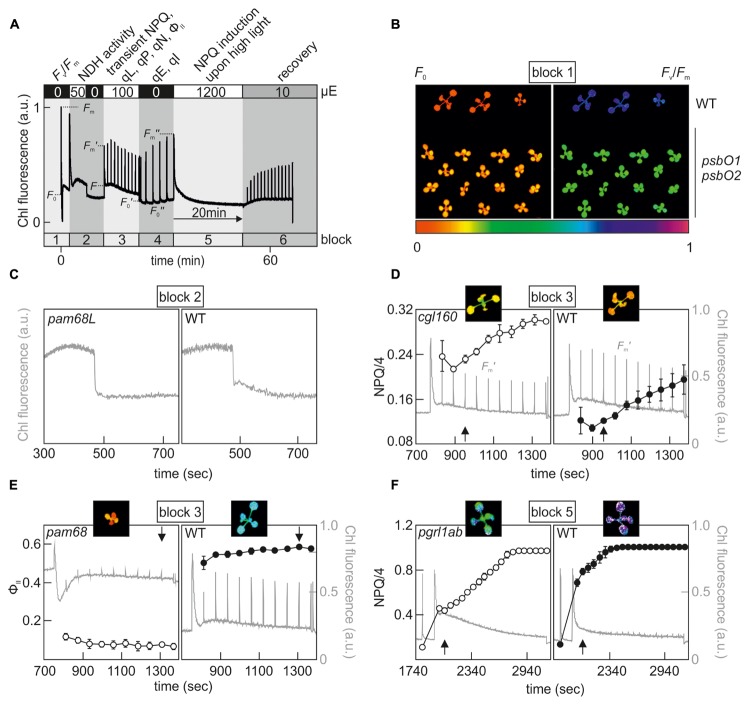
Example of a combined screening protocol based on Chl fluorescence video imaging which is able to identify *hcf, crr, npq, pam* and PSII repair mutants. **(A)** Chl fluorescence was monitored using an Imaging PAM system (Walz^®^) and the indicated sequence of actinic light conditions was executed in series to determine, in a single experimental run, the various photosynthetic parameters listed at the top. Plants were dark-adapted for 20 min and acclimated to measuring light for 5 min prior to the analysis. For further explanations, see the main text. *F*_v_/*F*_m_, maximum quantum yield of PSII; NPQ, non-photochemical quenching; qL, fraction of open PSII centers; qP, photochemical quenching coefficient; qN non-photochemical quenching coefficient; Φ_II_, effective quantum yield of PSII; qE, energy-dependent quenching; qI, photo-inhibitory quenching. **(B)** Example of an *hcf* mutant phenotype, which can be detected in measurement block 1. *F*_0_ and *F*_v_/*F*_m_ images of wild-type and *psbO1 psbO2* (Steinberger et al., 2015) Arabidopsis plants. **(C)** Identification of a *crr* mutant phenotype in block 2. Detail of the post-illumination fluorescence rise (PIF) analysis of *pam68L* (Armbruster et al., 2013), which is disrupted in NDH complex assembly. **(D)** Detection of a high NPQ phenotype in block 3. *F*_m_′ values were recorded every minute by applying saturating light pulses after a dark-light transition (100 μE m^-2^ s^-1^) and calculated NPQ values of the chloroplast ATP synthase-deficient mutant *cgl160* (Rühle et al., 2014) were compared to a wild-type control. **(E)** Example of a *pam* mutant phenotype, which can be distinguished at the end of block 3. Φ_II_ values of the PSII assembly mutant *pam68* (Armbruster et al., 2010) were compared to a wild-type control. **(F)** Detection of an *npq* phenotype in measurement block 5. NPQ analyses were carried out with the CEF mutant *pgrl1ab* (DalCorso et al., 2008) and compared to a wild-type control. False-color images for *F*_0_, *F*_v_/*F*_m_, NPQ/4, and Φ_II_ depicted at the time points highlighted by a black arrow represent values on a rainbow scale from 0 to 1 shown below **(B)**. Note that NPQ parameters in **(D,F)** are displayed in NPQ/4 to fit the standard color code ranging from 0 to 1. Chl fluorescence signals were normalized to *F*_m_ and are shown in gray on a scale from 0 to 1 in **(D–F)**.

In brief, a typical state-of-the-art CFVI analysis is based on the application of pulse-amplitude-modulated (PAM), measuring light (ML), which is generated by a powerful array of LEDs placed in a defined working distance to the sample. Those LEDs can also serve for the generation of short saturation pulses (SPs) and for actinic illumination (AL) of the samples to drive photosynthesis. Emitted red Chl fluorescence is detected by a computer-connected charge-coupled device (CCD) video camera which is protected from excitation light or near-infrared radiation by appropriate color glass filters. Custom software allows the conversion of Chl fluorescence signals into false color images, calculation of different photosynthetic parameters and quantitative analyses of the results. In general, plant or algal samples are dark-adapted prior to the measurements to open all PSII reaction centers. Then, samples are exposed to ML for dark fluorescence yield (*F*_0_) determination and to a short SP for maximum fluorescence yield (*F*_m_) measurement, respectively (see also **Figure 1A**). In this state, the PSII quantum yield (*F*_v_/*F*_m_) is maximal and can be calculated according to the equation *F*_v_/*F*_m_ = (*F*_m_-*F*_0_)/*F*_m_. AL is switched on and application of SPs provides maximum fluorescence yields (*F*_m_′) of illuminated samples. Effective PSII quantum yields (Φ_II_) are calculated by the equation Φ_II_ = (*F*_m_′-*F*)/*F*_m_′ (Genty et al., 1989), whereas the fluorescence yield (*F*) is recorded every time shortly before a SP and represents an average of several current fluorescence yield (*F*_t_) pictures. Electron transport rates through PSII [ETR(II)] at a given photosynthetically active radiation (PAR) can be calculated according to Schreiber et al. (1995), using the equation ETR(II) = Φ_II_ × PAR × 0.84 × 0.5. Maximum ETR(II) measured at saturating light intensity provides an estimate of the maximum photosynthesis rate (*P*_max_). *F*_m_′ values of illuminated samples are in general lowered compared to Fm by non-photochemical quenching (NPQ), which can be quantified according to the equation NPQ = (*F*_m_-*F*_m_′)/*F*_m_′ (Bilger and Björkman, 1990). NPQ mechanisms can be further examined in dark relaxation experiments. To this end, actinic light is switched off after a period of actinic light exposure and minimum fluorescence (*F*_0_^′′^) and maximum fluorescence yields (*F*_m_^′′^) are determined by application of SPs in the dark relaxation phase (**Figure 1A**). The two NPQ components qE (ΔpH-dependent feedback de-excitation, the major component of NPQ), and qI (photo-inhibitory quenching) can be calculated according to the equations qE = *F*_m_/*F*_m_′-*F*_m_/*F*_m_^′′^ (Thiele et al., 1997) and qI = (*F*_v_-*F*_v_^′′^)/*F*_v_ (Björkman and Demmig, 1987).

## Chl Fluorescence Video Imaging in Forward Genetic Approaches

### Screens for Chlamydomonas and Arabidopsis Mutants Defective in Photochemical Quenching

The first instance of the successful use of Chl fluorescence imaging to identify photosynthetic mutants was the detection of a ‘*high-chlorophyll-fluorescence*’ (*hcf*) phenotype in a population of methyl-methane sulfonate-mutagenized *Chlamydomonas reinhardtii* cells by Bennoun and Levine (1967) (**Table 1**). The screen was based on the fact that severe perturbations in photosynthetic electron transport, such as those caused by incubating algal cells in the presence of the photosynthetic electron transport inhibitor 3-(3,4-dichlorophenyl)-1,1-dimethylurea (DCMU), lead to high steady-state levels of Chl fluorescence. Following its application for screening of Chlamydomonas mutant libraries (see for example Harris, 1989) the concept was tested in higher plants (Miles and Daniel, 1973) and employed for the screening of maize (Miles and Daniel, 1974; Barkan et al., 1986; Taylor et al., 1987) and Arabidopsis mutant libraries (Dinkins et al., 1994; Meurer et al., 1996b). Several factors involved in chloroplast biogenesis were identified using the *hcf* phenotyping method, including the maize proteins HCF106, HCF60, and HCF136 (reviewed in: Belcher et al., 2015) and the Arabidopsis proteins HCF5 (Dinkins et al., 1997), HCF101 (Lezhneva et al., 2004), HCF107 (Felder et al., 2001), HCF109 (Meurer et al., 1996a), HCF145 (Lezhneva and Meurer, 2004; Manavski et al., 2015), HCF152 (Meierhoff et al., 2003) and LPA1 (Peng et al., 2006).

**Table 1:** Chronology of Chl fluorescence phenotyping based gene discovery studies.

Phenotype/principle	Organism	Authors
*hcf*	Chlamydomonas	Bennoun and Levine, 1967
*hcf*	Maize	Miles and Daniel, 1974
*hcf*	Maize	Barkan et al., 1986
*hcf*	Maize	Taylor et al., 1987
*hcf*	Arabidopsis	Dinkins et al., 1994
*hcf*	Arabidopsis	Meurer et al., 1996b
Low NPQ after high light treatment	Chlamydomonas	Niyogi et al., 1997
Low NPQ after high light treatment	Arabidopsis	Niyogi et al., 1998
Deficiency in state transition	Chlamydomonas	Fleischmann et al., 1999
Deficiency in state transition	Chlamydomonas	Kruse et al., 1999
Low NPQ after high light treatment	Arabidopsis	Shikanai et al., 1999
Alterations in Φ_II_	Arabidopsis	Varotto et al., 2000a
Lack of NDH complex activity	Arabidopsis	Hashimoto et al., 2003
Identification of mutants with unchanged Φ_II_ after acclimation to high light	Arabidopsis	Walters et al., 2003
Low NPQ after high light treatment	Arabidopsis	Kalituho et al., 2006
*hcf*	Arabidopsis	Peng et al., 2006
Identification of photorespiration mutants by comparison of *F*_v_/*F*_m_ values under varying CO_2_ concentrations	Arabidopsis	Badger et al., 2009
Identification of NDH complex mutants in a guilt-by-association approach	Arabidopsis	Takabayashi et al., 2009
Quantitative genetic analysis of thermal dissipation	Arabidopsis	Jung and Niyogi, 2009
Identification of CEF mutants with a high qE	Arabidopsis	Livingston et al., 2010
Affected Chl fluorescence transients	Chlamydomonas	Houille-Vernes et al., 2011
Affected Chl fluorescence transients	Chlamydomonas	Tolleter et al., 2011
Identification of hydrogenase-deficient mutants using Φ_II_ measurements under anaerobiosis	Chlamydomonas	Godaux et al., 2013
Suppressor screen of mutants with a high NPQ in the absence of PsbS	Arabidopsis	Brooks et al., 2013
Identification of mutants with altered mitochondrial respiration using *F*_v_/*F*_m_ measurements	Chlamydomonas	Massoz et al., 2015
Quantitative genetic analysis of variations in Φ_II_ acclimation to irradiance changes in different natural Arabidopsis accessions	Arabidopsis	van Rooijen et al., 2015
High transient NPQ after a dark-light shift	Arabidopsis	Zhang et al., 2016
Identification of mutants with emergent photosynthetic phenotypes under dynamic environmental conditions	Arabidopsis	Cruz et al., 2016
Identification of mitochondrial complex I mutants in a Φ_II_-based screening of a mutagenized *pgrl1* library	Chlamydomonas	Massoz et al., 2017
Identification of mutants affected in the slowly reversible photoprotective form of NPQ termed qH	Arabidopsis	Malnoë et al., 2017

*hcf, high chlorophyll fluorescence; NPQ, non-photochemical quenching; Φ_*II*_, effective quantum yield of PSII; NDH complex, NADH dehydrogenase-like complex; *F*_*v*_/*F*_*m*_, maximal quantum yield of PSII; qE, energy-dependent quenching; CEF, cyclic electron flow*.

Even though such *hcf* mutant screens can be performed rapidly and efficiently, and have significantly enhanced our knowledge of the molecular repertoire required for photosynthesis and chloroplast biogenesis, only mutants with severe defects can be unequivocally detected, and these are often lethal under photoautotrophic conditions. However, technological progress in Chl fluorescence analyses during the 1980s and 1990s allowed the technique to be employed for more elaborate modes of screening, and led to the identification of algal or plant mutants with relatively modest alterations in photosynthetic performance. For example, Varotto et al. (2000a,b) identified ‘*photosynthesis affected mutants*’ (*pam*) in Arabidopsis on the basis of their lower effective quantum yields (Φ_II_) (Genty et al., 1989) using a combination of a pulse-amplitude-modulation fluorometer (Schreiber et al., 1986) and an automated screening system. This set-up facilitated the screening of large *En* transposon or T-DNA mutagenized Arabidopsis populations, and *pam* mutants disrupted in the nucleus-encoded photosystem I subunits PsaE1 (*pam4*) (Varotto et al., 2000b) and PsaD1 (*pam62*) (Ihnatowicz et al., 2004), the metal-ion transporter IRT1 (*pam25*) (Varotto et al., 2002), and the cytoplasmic *N*-acetyltransferase AtMAK3 (*pam21*) (Pesaresi et al., 2003), as well as the PSII assembly factor PAM68 (*pam68*) (Armbruster et al., 2010), were isolated and functionally characterized in subsequent studies.

### Screens for Chlamydomonas and Arabidopsis Mutants Affected in Non-Photochemical Quenching (NPQ)

Due to their sessile lifestyle, many multicellular photosynthetic organisms have evolved various strategies to cope with light stress (Ort, 2001). When the photosynthetic machinery is exposed to excessively high levels of light, short- and long-term adaptive responses are triggered at the molecular level, which allow for the thermal dissipation of excited energy by NPQ mechanisms to prevent over-reduction of the electron transport chain. At least four processes contribute to NPQ: qE, qZ (zeaxanthin-dependent quenching), qT (state-transition-dependent quenching) and qI (reviewed in: Ruban, 2016). Several CFVI-based screens have been performed on mutagenized Chlamydomonas and Arabidopsis populations with the aim of dissecting the genetics of NPQ (**Table 1**). Mutant identification was essentially based on the comparison of two video images of Chl fluorescence captured under different illumination conditions. The first picture was taken in the dark-adapted state during a saturating light pulse (*F*_m_), or shortly after the onset of high-light treatment (*F*). The second image was recorded after several minutes of exposure to high light either during a saturating light pulse (*F*_m_′) or not (*F*′). The NPQ values derived using the equation (*F* -*F*′)/F′ or (*F*_m_-*F*_m_′)/*F*_m_′ were then visualized as false-color images, and several Chlamydomonas and Arabidopsis mutants affected in NPQ of excited Chl states could be identified. Subsequent analyses revealed three distinct groups of mutants with aberrant NPQ (reviewed in: Golan et al., 2004). Mutants in the first group were impaired in the generation of a proton gradient across the thylakoid membrane, which is a prerequisite for the induction of qE (the ΔpH-dependent quenching component of NPQ), and were consequently defined as ‘*proton gradient regulation’* mutants (*pgr*). One such mutant, *pgr1*, was further characterized, and shown to be defective in the photosynthetic electron transfer C (*PETC*) gene, which encodes the Rieske subunit of the cytochrome *b_6_f* complex (Munekage et al., 2001). Another mutant line impaired in the build-up of the proton gradient is *pgr5* (Shikanai et al., 1999; Munekage et al., 2002). It lacks a component of the antimycin A-sensitive cyclic electron flow (CEF) pathway, which is mediated by the ferredoxin-plastoquinone reductase PGRL1/PGR5 (Hertle et al., 2013). The second group with aberrant NPQ comprised the mutants *npq1* and *npq2*, which display defects in the xanthophyll cycle and are disrupted in the violaxanthin de-epoxidase and zeaxanthin epoxidase, respectively (Niyogi et al., 1997). The third type of mutant (*npq4*) showed normal pigment composition, xanthophyll cycle activity and photosynthetic electron transport, but this mutant was nevertheless specifically affected at the level of qE (Li et al., 2000). It turned out that the *npq4* mutant lacks the PSII-associated protein S (PsbS), which is now known to be the luminal pH sensor that triggers NPQ within the PSII antenna in plants (Li et al., 2000). In a subsequent study, which was designed to isolate Arabidopsis lines affected in other slowly reversible NPQ components, CFVI was used to screen mutagenized seedlings for suppressors of the *npq4* phenotype (Brooks et al., 2013). This screen yielded the *suppressor of quenching 1* (*soq1*), which has a high NPQ even in the absence of PsbS and lacks a thylakoid membrane protein (SOQ1) that harbors a thioredoxin-like, a β-propeller and a haloacid-dehalogenase domain. SOQ1 maintains light-harvesting efficiency and prevents formation of a slow, reversible NPQ mechanism that is independent of qE, qZ, and qT, but participates in a photoprotective, ‘qI-like’ mechanism termed qH (Malnoë et al., 2017). To identify factors involved in qH, suppressors of *soq1 npq4* were screened for by CFVI and two mutants affected either in chlorophyllide *a* oxygenase (CAO) or the plastid lipocalin (LCNP) showed a reversion to the low NPQ phenotype of *npq4*. In-depth analyses of both mutants provided evidence that qH operates under high-light and cold stress, and can be localized to the peripheral LHCII antenna of PSII and requires LCNP (Malnoë et al., 2017).

A further step toward an understanding of the molecular basis of NPQ was the identification of so-called ‘*state transition*’ (*stt*) mutants with alterations in qT. State transitions involve the reversible association of the mobile pool of light-harvesting-complex II proteins (LHCIIs) with either PSII (state 1) or PSI (state 2) and re-establish a balanced distribution of light energy between the photosystems. Several studies (Fleischmann et al., 1999; Kruse et al., 1999) took advantage of the fact that the green alga Chlamydomonas undergoes large changes in Chl fluorescence during state transitions, which can be attributed to its significantly higher fraction of mobile LHCIIs (about 80%, Delosme et al., 1996) compared to land plants (15–20%) (Allen, 1992). Mutants affected in state transitions were identified by comparing fluorescence images taken under state-1 and state-2 conditions, and this type of differential fluorescence screen enabled Fleischmann et al. (1999) to isolate four *stt* mutants (*stt2, stt3, stt5*, and *stt7*). These mutants were characterized by high Chl fluorescence levels at room temperature even under state-2 conditions, indicating that they were physiologically locked in state 1. Further analyses showed that *stt7* lacks the thylakoid serine-threonine protein kinase Stt7, which is required for phosphorylation of LHCII in response to state-2 conditions (Depège et al., 2003).

### Screening for Chlororespiratory Arabidopsis Mutants

In addition to the predominant linear electron flow pathway, which results in the production of both NADPH and ATP, two CEF routes around PSI have been described that are important for balancing the ATP/NADPH budget of photosynthesis, as well as for protecting the photosystems from photodamage in plants (reviewed in: Yamori and Shikanai, 2016). One of these pathways is mediated by the NADH-like dehydrogenase (NDH) complex, which is also responsible for chlororespiration in the dark. To identify ‘*chlororespiratory reduction*’ mutants (*crr*) which are disrupted in NDH function, Hashimoto et al. (2003) established a CFVI-based screening system, in which the post-illumination rise of Chl fluorescence (PIF) after a low-light treatment was monitored in an Arabidopsis mutant population. Under such conditions, the fluorescence signal is almost proportional to the reduction state of the plastoquinone pool (Krause and Weis, 1991), so that the chlororespiratory activity of the NDH complex can be derived from the degree to which the PIF is depressed in mutants with a dysfunctional NDH complex (Shikanai et al., 1998). Screening of over 50,000 M2 seedlings for aberrant PIFs led to the identification of 17 *crr* mutants in Arabidopsis. These could be assigned to at least 11 loci, and further analyses revealed the existence of novel NDH subunits and allowed the functional characterization of factors required for efficient NDH complex biogenesis (reviewed in: Peng et al., 2011).

### Screening for Arabidopsis Mutants Affected in Acclimation of Photosynthesis to the Environment

Plants and algae can undergo photosynthetic acclimation processes which take place over periods of hours or days and entail substantial changes in plastid and nuclear gene expression, as well as adjustments of the photosynthetic apparatus. For instance, the long-term response to high light levels has been thoroughly studied and, instead of reducing the demands on light harvesting, it actually enhances the capacity for electron transport and carbon dioxide fixation. To investigate the molecular mechanisms behind the signal cascades that activate the acclimation response to high light, Walters et al. (2003) screened an Arabidopsis mutant population for alterations in ‘*acclimation of photosynthesis to the environment*’ (*ape*). Their CFVI screen was based on the observation that in wild-type Arabidopsis plants a 3-day exposure to high light raises effective quantum yields (Φ_II_), and its goal was to identify mutants that were unable to increase *P*_max_ under these conditions. Among the three *ape* mutants obtained, which showed distinct acclimation-defective phenotypes, *ape2* exhibited a lower *P*_max_ under all light regimes, and was disrupted in the chloroplast envelope triose-phosphate/phosphate translocator (TPT). Subsequent studies using Arabidopsis double and triple mutants altered in the day and night modes of photoassimilate export from the chloroplast provided evidence that carbohydrates act as chloroplast-to-nucleus retrograde signals and modulate the acclimation response to high light (Schmitz et al., 2012, 2014).

### Screening for Arabidopsis Mutants Affected in Photorespiration

Ribulose-1,5-bisphosphate carboxylase/oxygenase (RuBisCO) not only fixes atmospheric CO_2_ but also oxygen. The phosphoglycolate generated by the latter reaction must be degraded via a complex mechanism which is known as photorespiration, because CO_2_ is released during the process. The photorespiratory pathway is distributed between four compartments (chloroplasts, cytosol, peroxisomes and mitochondria) and requires the action of several transporters and enzymes. Most of the early mutants affected in photorespiration were identified by their ability to grow normally in a high concentration (1%) of CO_2_, while becoming chlorotic when shifted to ambient air (Somerville, 1986). Recently, it was shown that mutations in components involved in the photorespiratory pathway also impair photosynthetic light reactions, as revealed by the observation that photorespiratory mutants transferred from high to ambient CO_2_ concentrations showed a decline in PSII functionality (Takahashi et al., 2007). Thus, Badger et al. (2009) set up a CFVI screen designed to detect mutants with more subtle photorespiratory phenotypes. To this end, levels of PSII function in mutagenized Arabidopsis seedlings grown under high concentrations of CO_2_, and in its absence, were compared. Two major mutant phenotype classes could be distinguished. One group comprised ‘photorespiration-like’ mutants, which were characterized by *F*_v_/*F*_m_ values that were close to those of wild-type plants under high CO_2_ concentrations, but significantly lower than normal in the absence of CO_2_. The second group consisted of lines in which *F*_v_/*F*_m_ values were depressed even under high concentrations of CO_2_. Remarkably, some members of the second group were able to partially recover PSII functionality after exposure to zero CO_2_ concentrations, and were therefore named for this ‘reverse photorespiration’ phenotype (Badger et al., 2009).

### Screening for Chlamydomonas Mutants Impaired in Hydrogenase Activity

As aquatic organisms, many unicellular green algae are characterized by a remarkably flexible metabolism, and can acclimate rapidly to anaerobic conditions (Terashima et al., 2010; Grossman et al., 2011). As part of an extensive response to anaerobiosis, expression and synthesis of oxygen-labile [Fe–Fe] hydrogenases are induced in *C. reinhardtii* and hydrogen production is linked to photosynthesis by ferredoxin-mediated electron supply. Several factors required for expression, maturation and activity of [Fe–Fe] hydrogenases have been identified, most of them through a H_2_-sensing, chemochromic screening system that can discriminate Chlamydomonas mutants with aberrant H_2_ production capacities (reviewed in: Hemschemeier et al., 2009). An alternative, less time-consuming approach has been demonstrated by Godaux et al. (2013), and takes advantage of the observation that mutants with defects in [Fe–Fe] hydrogenase activity exhibit low effective PSII quantum yields shortly after a shift from dark anaerobiosis to saturating light conditions. As a proof of concept, screening of a small Chlamydomonas population of about 3000 strains generated by insertional mutagenesis yielded five mutants with a Chl fluorescence signature similar to that of the [Fe–Fe] hydrogenase-deficient control strain, and one of them turned out to be defective in the previously characterized [Fe–Fe] hydrogenase assembly factor G (HydG) (Posewitz et al., 2004). Moreover, in various mutants affected in anaerobic energy metabolism, the effective quantum yield of PSII was shown to be correlated with the level of [Fe–Fe] hydrogenase activity. Thus, the screening system represents a time-saving, alternative approach to the chemochromic method, and is capable of detecting mutants impaired in [Fe–Fe] hydrogenase biogenesis, regulation or activity.

### Screening for Chlamydomonas Mutants Altered in Mitochondrial Respiration

Although respiration and photosynthesis take place in different organelles in photosynthetic eukaryotes, the energy metabolisms of mitochondria and chloroplasts are intertwined at multiple levels. Not only do these organelles share over 100 dual-targeted proteins (reviewed in: Carrie and Small, 2013), provide both ATP and contribute to photorespiration, chloroplasts can shuttle reducing power to mitochondria via the malate valve (reviewed in: Scheibe, 2004; Kramer and Evans, 2011). Functional cooperation between mitochondria and chloroplasts in balancing the cellular ATP/NADPH ratio becomes even more obvious when compensatory acclimation processes are studied in mutants affected in photosynthesis or respiration. For instance, in Chlamydomonas mutants defective in different complexes of the respiratory electron transport chain, the resulting ATP deficiency is counterbalanced by increased non-photochemical reduction of the plastoquinone pool mediated by the chlororespiratory pathway, LHCII protein association to PSI and cyclic photophosphorylation (Cardol et al., 2003). Furthermore, the Chlamydomonas strain *pgrl1* disrupted in the proton regulation 5 like 1 protein (PGRL1), which was identified as a CEF mutant in CFVI-based screen (Tolleter et al., 2011), compensates for ATP deficiency by increasing oxygen photoreduction downstream of PSI and shows higher susceptibility to mitochondrial inhibitors (Dang et al., 2014). These results are consistent with the finding that overall fitness and yields of photosynthesis were only significantly reduced when state transitions and mitochondrial respiration were concomitantly impaired in the Chlamydomonas double mutant *stt7-9 dum22* (Cardol et al., 2009). Thus, increased cyclic electron transport rates induced by state 2 transitions can supply extra ATP when respiratory ATP production becomes limiting and, conversely, mitochondrial cooperation is increased when CEF is downregulated in Chlamydomonas. One important conclusion that could be drawn from these studies was that PSII efficiencies were reduced in respiratory mutants and was explained by enhanced rates of non-photochemical reduction of plastoquinone mediated by the chlororespiratory pathway and preferential association of LHCII proteins with PSI (Cardol et al., 2003). Massoz et al. (2015) therefore used *F*_v_/*F*_m_ values as an initial criterion to select mutants affected in mitochondrial respiration. Several mutants disrupted in subunits of the respiratory complex I or the isocitrate lyase were isolated from a collection of about 2900 insertional mutants generated in either a wild-type or a state transition-defective strain (*stt7-9*). A later refinement of the screening procedure used the CEF mutant *pgrl1* as the starting strain with a view to isolating mutants impaired in mitochondrial complex I (Massoz et al., 2017). As proof of concept, the double mutant *pgrl1*Δ*nd4*, which is deficient in both CEF and complex 1, was generated and shown to exhibit a lower PSII efficiency than either of the single mutants. Subsequent screening of about 3000 insertional mutants created in the *pgrl1* background resulted in 46 mutants with reduced PSII efficiency, of which three were complex I mutants. Further analyses revealed that one of these was disrupted in NADH dehydrogenase [ubiquinone] 1 alpha subcomplex assembly factor 3 (NDUFAF3), a complex I assembly factor also conserved in humans (Massoz et al., 2017).

## Chl Fluorescence Video Imaging in Reverse Genetic Approaches

Forward genetic approaches still dominated mutant searches in the late 1990s and early 2000s (Lloyd and Meinke, 2012), but thanks to advances in genome sequencing technologies, the establishment of large mutant libraries and the development of new genetic tools such as RNA silencing techniques (Mohr et al., 2014), and more recently genome editing tools (Yin et al., 2017), reverse genetics has since come to the fore. Indeed, in conjunction with the tremendous rise in the availability of myriad ‘omics’ datasets, reverse genetic strategies have become the more practicable choice, since laborious screens of large mutant libraries are circumvented and the underlying genetic defects are already known. Relative to classical forward genetic approaches, reverse genetic screens start with a significantly reduced number of lines or strains, which are generally disrupted in genes with poorly characterized or unknown functions. Depending on the stringency of preselection criteria (e.g., coregulation or phylogenomic studies), ‘the starting material’ can be narrowed down to a reasonable number of candidates which is compatible with the complexity of the required screening procedure. One example for the power of such ‘guilt-by-association’ approaches is the identification of three subunits of the NDH complex – NDF1, NDF2, and NDF4 (Takabayashi et al., 2009) now called photosynthetic NDH subcomplex B subunit PnsB1, PnsB2, and PnsB3 (Ifuku et al., 2011). In that study, genes of unknown function were selected on the basis of their co-expression with nucleus-encoded NDH subunits L, N, and O (NDHL, NDHN, and NDHO). In addition, Arabidopsis genes (of unknown function) were considered together with homologs found in cyanobacteria but not in green algae, since *C. reinhardtii* lacks a plant-type NDH complex. Insertion lines were identified for 21 of the 36 genes pre-selected by means of the bioinformatics screen, and these were tested for NDH activity. Remarkably, four of them (nearly 20%) failed to exhibit the post-illumination rise in fluorescence. Further studies provided evidence that the respective genes indeed code for the NDH subunits NDF1 (PnsB1), NDF2 (PnsB2), and NDF4 (PnsB3), whereas NDF3 corresponds to the chlororespiratory reduction protein 6 (CRR6), which is involved in NDH subcomplex A assembly (Munshi et al., 2006).

### Chl Fluorescence Image Analyses in Combined Screening Protocols

Since Chl fluorescence-based phenotyping is no longer as time-consuming as it once was, and manageable numbers of candidates can be examined in reverse genetics projects, contemporary screening approaches can be extended to more elaborate protocols in which subtle or multiple photosynthetic phenotypes can be detected in a single, albeit longer, experimental run. Commercial Chl video imaging systems now make it possible to set up automated measuring routines composed of several analytical blocks that can last for days. One example of such a combined screening protocol is shown in **Figure 1**, which we use routinely for initial phenotyping of selected Arabidopsis mutant lines.

In principle, the approach comprises six phases, in which most of the previously described Chl fluorescence signatures of photosynthetic mutants can be identified (**Figure 1A**). In the first block, the *F*_v_/*F*_m_ measurement allows one to assess PSII functionality and pinpoint mutants with an *hcf* phenotype, such as the Arabidopsis PSII subunit O (PsbO) knockdown mutant *psbO1 psbO2* (**Figure 1B**) (Steinberger et al., 2015). The second analytical block was designed to identify mutant lines with a *crr* phenotype, and detects NDH activity by means of a PIF measurement (**Figure 1C**). Block 3 implements a standard slow induction experiment, which is carried out under moderate actinic light intensities. Several informative parameters can be extracted in block 3 which reveal aspects of the transient dynamics of photosynthesis upon a dark-light shift. For instance, *pgr* mutants can be already identified at this stage by their low transient NPQ phenotype (DalCorso et al., 2008). Conversely, mutants affected in chloroplast F_1_F_0_-ATP synthase activity can be identified on the basis of their high NPQ (**Figure 1D**) (Rühle et al., 2014; Grahl et al., 2016; Zhang et al., 2016). The increased NPQ in such mutants can be attributed to a high operating qE, which is established as a result of proton accumulation in the thylakoid lumen already under moderate light intensities. Parameters determined at the end of block 3 reflect photosynthetic performance in the steady state, and an analysis of effective quantum yields (Φ_II_) uncovers *pam* mutants (**Figure 1E**). Samples in block 4 are shifted back into the dark and NPQ relaxation kinetics provide values of qI and qE, which was recently determined in an initial screening step to identify ‘*high cyclic electron flow around PSI*’ (*hcef)* mutants with altered CEF (Livingston et al., 2010). Block 5 also implements a dark-light shift experiment, but using excessive light intensities (1200 μE m^-2^ s^-1^) and longer exposure times (20 min), which allow the detection of *npq* mutants due to their aberrant NPQ induction patterns under high light (Niyogi et al., 1998). After the photodamage-inducing high-light treatment in block 5, the protocol ends with a recovery phase from high light (block 6) under low light intensities (10 μE m^-2^ s^-1^) and was designed to pick up mutants that are defective in the PSII repair mechanism (Schroda et al., 1999; Malnoe et al., 2014). Overall, the CFVI protocol outlined above can already uncover a wide range of phenotypes, but can be further expanded to cover a larger collection of photosynthetic parameters, such as the determination of *P*_max_ from light saturation curves (van Rooijen et al., 2015) and measurements of qT in state transitions (Pribil et al., 2010) or effective quantum yields/NPQ parameters under fluctuating light conditions (Cruz et al., 2016).

### Chl Fluorescence Image Phenotyping under Simulated Environmental Conditions

The photosynthetic lifestyle of plants and algae requires a high degree of flexibility and the ability to adapt to rapidly fluctuating environments. However, for reasons of scalability and reproducibility most of the screening studies referred to here were conducted with small Arabidopsis plants or algae grown in stable, standardized laboratory settings and would have been impossible with fully developed crops under field conditions. Furthermore, phenotyping of mutant collections involved measurements of only one or a few photosynthetic parameters, which were determined at one or more time points, thus providing a rather static picture of the highly dynamic process of photosynthesis. It is therefore obvious that many factors that contribute to the fine tuning of photosynthesis in response to dynamic environmental changes will have not been identified by previous screening procedures (Cruz et al., 2016). This assertion is also supported by the observation that plant lines lacking PsbS (Külheim et al., 2002), the LHCII serine/threonine-protein kinase STN7 (Grieco et al., 2012) or PGR5 (Suorsa et al., 2012) showed higher levels of photodamage and notable reductions in their growth rates (or lethality) only under fluctuating light conditions that were not observed under unchanging conditions. One logical and straightforward way to bypass this limitation would be to carry out phenotyping of mutant collections in the field, and suitable large-scale Chl fluorescence image analyzers are now available for this task (e.g., Field Scanalyzer) (Virlet et al., 2017). However, besides the fact that in several countries the cultivation of genetically modified plants in the field is either prohibited or subject to legal restrictions, such studies are complicated by a multitude of overlapping, unpredictable abiotic and biotic stress factors, and statistical evaluation of the results become particularly challenging. For these reasons, the dynamic environmental photosynthesis imager (DEPI) platform was developed for replication of natural, fluctuating growth conditions in the laboratory (Cruz et al., 2016). Several parameters can be controlled (light intensity, CO_2_ concentration, humidity and temperature) in the growth chamber, and rapid responses as well as long-term acclimation processes of photosynthesis can be assessed *in situ* by the integrated CFVI system in more than two hundred plants simultaneously. As a proof of concept, a library of over 300 T-DNA Arabidopsis lines disrupted in nuclear genes coding for chloroplast-targeted proteins (Ajjawi et al., 2010) was exposed to a 5-day regime of fluctuating light levels and screened for alterations in photosynthetic performance. As a result, *psb33* plants lacking PSII protein 33 (PSB33) (Fristedt et al., 2015) and several other conditional mutant lines showed transient, spatiotemporal-dependent phenotypes which could not be detected or were not reliably expressed under standard growth conditions. PSB33 is a green-lineage-specific protein (Merchant et al., 2007) predominantly found in non-appressed thylakoids of Arabidopsis chloroplasts and sustains D1 of PSII under fluctuating light conditions (Fristedt et al., 2017). Thus, the DEPI system can reveal new, complex and previously unseen phenotypes, and provides a versatile experimental platform with which to identify factors required for remodeling and regulation of photosynthesis under dynamic environmental conditions.

## Chl Fluorescence Video Imaging in Quantitative Genetic Approaches

Forward and reverse genetics are efficient strategies for elucidating the functions of a single gene or of small gene families, but these approaches reach their limits when the genetic architecture of a quantitative trait and its interaction with the environment needs to be determined. Most agronomically important traits (e.g., grain yield, grain size, ripening or flowering time) are controlled by multiple genes which have to be analyzed by quantitative genetic approaches, such as classical linkage mapping or genome-wide association studies (GWAS) (reviewed in: Bazakos et al., 2017). Natural variation also exists for photosynthetic traits and can be roughly divided into morphological and physiological variations, which have been investigated in several studies with different plant species (reviewed in: Flood et al., 2011). For instance, Jung and Niyogi (2009) examined natural NPQ variation in different Arabidopsis accessions and provided evidence that thermal dissipation is a quantitative trait that depends on multiple, nucleus-encoded genetic factors. Two high-NPQ QTLs (*HQE1* and *HQE2*) were identified in a quantitative trait locus (QTL) analysis which was performed with a F2 mapping population generated from a cross between a low-NPQ and a high-NPQ Arabidopsis accession (Jung and Niyogi, 2009). Remarkably, *HQE1* and *HQE2* were not mapped to previously characterized factors identified in forward genetic approaches, indicating that quantitative genetics can serve as a complementary strategy to dissect the genetic architecture of thermal dissipation.

Even though quantitative genetic approaches have a long history in plant science, their potential for photosynthesis research has not yet been fully explored. This may simply reflect the high complexity of the genetic architecture of photosynthesis, which not only comprises the several hundred genes directly involved in biogenesis processes, regulation or acclimation of photosynthesis, but also involves two quite distinct genetic systems (plastid and nuclear genome) with different inheritance modes. Moreover, successful quantitative genetic approaches in photosynthesis research require reproducible, non-invasive, high-throughput phenotyping pipelines that were not available until recently. However, several platforms have been developed in recent years. Examples include FluorImager (Barbagallo et al., 2003), GROWSCREEN FLUORO (Jansen et al., 2009), PlantScreen (Humplík et al., 2015), Phenovator (Flood et al., 2016), the DEPI system (Cruz et al., 2016) or the crop population growth information detection system (Wang et al., 2017), which now integrate CFVI analyses into their phenotyping facilities. As an example for the combination of a GWAS and a high-throughput Chl fluorescence phenotyping approach, van Rooijen et al. (2015) have explored the natural genetic variation for acclimation of photosynthetic light use efficiency (Φ_II_) in 344 Arabidopsis accessions. Of 63 newly identified gene candidates, 13 encode chloroplast-localized proteins, most of which are either associated with abiotic stress responses or have unknown functions.

## Gene Discovery Through Chlorophyll Fluorescence Video Imaging – a Brief Outlook

Due to the advances in imaging and data acquisition technologies in the last two decades, Chl fluorescence-based analyses have entered the ‘phenomics’ field and promise to increase our knowledge of photosynthesis substantially. Modern phenotyping systems are highly flexible and will allow the identification of new genotype-phenotype-environment relationships and accelerate gene discovery studies significantly.

The next obvious step will be to determine photosynthetic parameters of large-scale, indexed mutant collections like the Arabidopsis unimutant (O’Malley and Ecker, 2010), the Arabidopsis Chloroplast 2010 Project (Ajjawi et al., 2010) and the GABI-DUPLO double mutant (Bolle et al., 2013) collections, or the recently generated Chlamydomonas mutant collection CLiP (Li et al., 2016). Consequently, with the exception of screening approaches under highly specialized conditions, tedious forward genetic screening procedures, which were carried out by single researchers or research groups in the past, will become obsolete. A major task in the future will lie in the processing, handling, quality control, maintenance, storage, analysis and sharing of the vast amount of data collected by CFVI-based phenotyping studies, which will become even more challenging when such screens are combined with other non-invasive phenotyping technologies (Walter et al., 2015; Tardieu et al., 2017). But computational techniques for assessing the quality of phenotypic data (Xu et al., 2015) and analyzing massive amounts of data in order to reveal dynamic relationships between phenotypes and environment (Yang et al., 2017) have already been developed, and these will eventually replace manual evaluation methods.

Chl fluorescence video imaging also has the potential to be an important technological driver in crop science, since it offers an efficient screening technology for rapid evaluation of plant performance under stress conditions such as drought, salinity, freezing, chilling, high temperature or nutrient deficiency (reviewed in: Baker and Rosenqvist, 2004). CFVI is of particular interest in plant breeding programs, since low-cost and precise high-throughput phenotyping technologies have been regarded as one of the major bottleneck in the postgenomic era of plant breeding (reviewed in: Araus and Cairns, 2014). A further challenge is the difficulty to extrapolate results gained under a strictly controlled environment (such as a growth chamber or greenhouse) to field conditions. It is therefore inevitable to establish high throughput phenotyping technologies under heterogeneous field conditions to analyze quantitative traits and to elucidate their underlying genetic architecture for future breeding efforts. Significant progress in non-invasive sensor and imaging technology has been made (reviewed in: White et al., 2012; Fiorani and Schurr, 2013) and the Field Scanalyzer system installed at Rothamsted Research (United Kingdom) by LemnaTec GmbH (Germany) is one example, which now employs Chl fluorescence based measurements for high-throughput phenotyping in the field (Virlet et al., 2017).

While recent work has mainly focused on scaling up CFVI screening systems for simultaneous evaluation of large sample collections, a future direction might be to explore the potential of screening single cells by exploiting their Chl fluorescence fingerprints. Flow cytometry technologies are well established for unicellular microalgae in environmental and toxicological studies (reviewed in: Hyka et al., 2013) and several flow cytometry studies with plant protoplasts have been reported (Harkins et al., 1990; Galbraith, 2007; Berendzen et al., 2012; You et al., 2015). Flow cytometry is generally coupled to fluorescence-activated cell sorting, which permits the isolation of a desired cell population with specific physiological properties. Recently, this technique has been successfully employed to screen high-lipid Chlamydomonas mutants that were stained with the lipid-sensitive dye Nile Red prior to screening (Xie et al., 2014; Terashima et al., 2015) or to identify protein-protein interactions in plant protoplasts by combining bimolecular fluorescence complementation with flow cytometry (Berendzen et al., 2012). Moreover, Chl autofluorescence has been used in flow cytometry studies as an endogenous probe to sort tobacco mesophyll protoplasts (Harkins et al., 1990) and to discriminate between different phytoplankton species by cytometric approaches (Hildebrand et al., 2016). Although implementation will be challenging, the combination of flow cytometry and Chl fluorescence kinetics-based cell sorting can provide a fast means of screening mutagenized cell populations for specific Chl fluorescence phenotypes.

## Author Contributions

TR designed and wrote the article. BR and DL wrote the article.

## Conflict of Interest Statement

The authors declare that the research was conducted in the absence of any commercial or financial relationships that could be construed as a potential conflict of interest.
